# Training promotores to lead virtual hereditary breast cancer education sessions for Spanish-speaking individuals of Latin American heritage in California

**DOI:** 10.1186/s12905-022-01902-y

**Published:** 2022-08-08

**Authors:** Micaela Reyna, Rebeca Almeida, Alejandra Lopez-Macha, Shannon Fuller, Ysabel Duron, Laura Fejerman

**Affiliations:** 1 San Juan Bautista School of Medicine, Caguas, Puerto Rico; 2 California Polytechnic State University, San Luis Obispo, CA USA; 3 David Geffen School of Medicine, University of California Los Angeles, Los Angeles, CA USA; 4 Division of Prevention Sciences and Center for AIDS Prevention Studies, University of California San Francisco, San Francisco, CA USA; 5 The Latino Cancer Institute, San Jose, CA USA; 6 Department of Public Health Sciences and Comprehensive Cancer Center, University of California Davis, 451 Health Sciences Dr., Davis, CA 95616 USA

**Keywords:** Preventative medicine, Health education training, Hereditary breast cancer, Virtual education prevention, Qualitative research

## Abstract

**Background:**

Awareness about hereditary breast cancer and the preventative steps to minimize disease risk is lower in Hispanic/Latina individuals than non-Hispanic White women in the United States. For this reason, we developed a promotor-based hereditary breast cancer education and risk identification program for self-identified Hispanic/Latina women, which included training promotores in basic genetics and hereditary breast cancer. This study explored promotores’ experiences receiving training and participating in virtual practice sessions as well as changes in knowledge about hereditary breast cancer.

**Methods:**

A total of ten promotores underwent a two-week basic training led by the promotores organization and an eight-hour in person hereditary breast cancer training workshop. Demographic information along with pre- and post-training surveys were completed by ten promotores who participated in the training workshop. Surveys were given to determine changes in knowledge of hereditary breast cancer and genetics. Of the ten promotores, two were selected to lead community education sessions and participated in 6 semi-structured interviews. All interviews and practice sessions were conducted using a virtual platform.

**Results:**

The data revealed that after the 8-h workshop and practice sessions, promotores felt confident about their ability to conduct virtual education sessions with the community. Interviews identified key facilitators to success such as a supportive environment, practice presentations, and personal motivation. Learning the online platform was considered the biggest challenge by the promotores, as opposed to learning complex genetics topics.

**Conclusions:**

These results provide further evidence supporting promotores’ willingness and ability to provide health education on relatively complex topics. It also offers insight into the challenges of presenting information to vulnerable populations using an online platform and the additional support that is required to ensure a positive outcome.

**Supplementary Information:**

The online version contains supplementary material available at 10.1186/s12905-022-01902-y.

## Introduction

Among women of Latin American heritage in the United States (U.S.), invasive breast cancer is the leading cause of cancer death and 3100 deaths are predicted to occur in 2021 [1]. Inherited genetic mutations such as BRCA 1 and BRCA 2 account for 5–10% of all breast cancer cases and individuals with a BRCA mutation have a 69–72% chance of developing breast cancer by age 80 [2, 3]. Preventive measures are effective in decreasing breast cancer mortality especially among individuals with BRCA mutations who have an increased cancer development risk [4, 5]. However, Hispanic/Latina (H/L) women with BRCA 1 and BRCA 2 mutations are less likely to undergo risk-reducing surgeries and cancer screening compared to Non-Hispanic White (NHW) women [6]. H/L are also more likely to have advanced-stage cancer compared to NHW women [6, 7].

There are multiple barriers H/L face when accessing health information and care, including health literacy [8–13]. Lack or limited health insurance coverage and low socioeconomic status are additional factors that disproportionately affect H/L women and contribute to worse breast cancer outcomes [8, 9, 14]. In 2020, H/L had the highest uninsured rate of any demographic at 18.3% and H/L had a poverty rate of 17.0% which is more than twice the rate for NHW populations [15, 16]. Lack of insurance may discourage H/L from seeking and receiving preventative medical care, and potentially lead to adverse health outcomes [17, 18].

Stakeholder-informed programs are crucial to addressing barriers to care and disparities in breast cancer outcomes [19]. One promising approach involves partnering with promotores (Spanish term for community healthcare educators/workers) to share preventive education about health and build a stronger connection between healthcare systems and vulnerable populations [19]. Promotores-led approaches have been successful in addressing other health-related topics such as chronic diseases and cancer screening, as well as changing lifestyle behaviors [20–27]. For these reasons our program, ‘Tu Historia Cuenta’, was developed by The Latino Cancer Institute (TLCI) in collaboration with academic partners at the University of California San Francisco (UCSF) and Davis [28]. The goal of this promotores-led education and risk identification program is to increase awareness about breast cancer, particularly HBC, among the Spanish-speaking H/L community in California [28]. Additionally, the program identifies women with a strong breast cancer family history who could benefit from genetic counseling and testing. To our knowledge, this is the first Spanish language HBC education program targeting the H/L community in California that emphasizes the understanding of genetic concepts. This program is different from other programs aiming to educate promotores on HBC as it provides training on content, educational materials, and instruction on how to use the materials in the community [29].

Promotores-led education programs have been shown to increase screening practices among H/L which is why it is important to continue improving the approaches used to train these valuable community educators, particularly when using non-traditional approaches such as virtual training [27].

This study aimed to assess the promotores’ experience participating in ‘Tu Historia Cuenta’ program training and explore their confidence and ability to share accurate HBC knowledge. Due to the complexity of HBC and the lack of online education interventions that consider HBC genetics, it was important to evaluate the promotores’ acceptance and understanding of the materials to improve the program. A similar study performed in South Florida demonstrated that assessing promotores’ knowledge and understanding showed how promotores-led approaches are effective and highlighted ways to improve them [30]. This provides additional evidence as to why capturing promotores’ experience in a HBC education program is important in the California community. These experiences and recommendations can also inform other community health programs targeting H/L populations.

## Materials and methods

### Study design

The goal of this study was to evaluate promotores’ ability and confidence to explain important HBC concepts to community members and understand the training experience from the promotores’ perspective. This was designed as a qualitative study to capture the experience of promotores who were recruited to implement the ‘Tu Historia Cuenta’ program. Additionally, it included a short survey to describe promotores’ basic demographics and evaluate pre- and post-workshop training responses for an original cohort of promotores from which the program leaders where recruited.

Due to the COVID-19 pandemic, the HBC program moved to an online platform and the promotores performed virtual practice sessions. Qualitative interviews with promotores were conducted before and after each practice session to gain a detailed understanding of promotores’ self-perception of their ability to inform the community about HBC.

### Recruitment and eligibility criteria

A community-based promotores organization, partnered with researchers at UCSF to recruit promotores for the “Tu Historia Cuenta” program. Outreach to recruit promotores was focused on individuals who resided in the city of Sacramento and San Francisco as these were the targeted areas for the program. The research team required that recruited promotores had not been exposed to HBC prevention education materials in the past.

A group of ten participants underwent a two-week basic training led by the promotores organization and an eight-hour in person HBC training workshop conducted by the research team. The goal of the two-week basic training was to improve the promotores' knowledge and leadership skills. This in-person training included six modules covering topics such as, promotores role, effective communication, conflict resolution, non-formal learning, learning styles, and ethics. Each module was taught in eight-hours for a total of 48 hours during the span of two workweeks. The one day eight-hour HBC training presented the HBC information to the promotores in a simple manner while clarifying any questions the promotores might have had [28].

The program’s budget limited recruitment to four promotores to serve the San Francisco and Sacramento areas. The promotores were chosen based on flexible work schedule, previous community workshop experience, and residence near the area being served. The goal of the hiring process was to select promotores with the greatest potential to positively impact the H/L community. At the start of the program in March 2020, two participants left the project due to COVID-19 related reasons. The qualitative portion of the study is based on the additional training experience of two promotores who were actively preparing for virtual community presentations.

### Data collection and analysis

#### Survey

Demographic information and pre- and post-training surveys were completed by ten promotores who participated in the HBC training workshop. Surveys given before and after training sessions were administered to determine changes in knowledge of HBC and genetics. Both surveys consisted of the same 16 basic level questions about breast cancer and genetics [28]. Promotores’ responses for both surveys were examined individually and compared to assess the training’s impact on performance. Survey data was analyzed by looking at improvement in the number of questions answered correctly in the post-workshop survey which we considered suggestive of HBC knowledge improvement.

#### Practice sessions

Virtual practice education sessions were conducted during an 8-week period before implementation to allow promotores a space to enhance their HBC presentations before engaging with the community. Each of the participants presented to the research team and colleagues as if they were doing a session with community members. Following the exercise, the promotores were provided feedback to enhance their presentation for the next session. Each promotor led a presentation three times and presentations lasted up to three hours per practice session. Before and after every practice trial, the promotores were interviewed to capture their experience with the HBC material.

#### Interviews

Semi-structured interviews were conducted via telecommunication (i.e., Zoom) due to the COVID-19 pandemic. Separate guides were developed for pre- and post-session interviews to gather promotores’ perspectives on what concepts within the HBC education program were challenging and self-perceptions of their practice presentation. The two promotores in the study were followed for a period of eight weeks to observe their progress presenting HBC topics (refer to the Additional file 1 for interview questions).

The promotores were interviewed before and after each HBC virtual practice education event for a total of 12 interviews, six interviews per person. Each session lasted between 20 and 35 minutes. Participants provided verbal informed consent and all interviews were conducted in Spanish by a bilingual researcher. Interviews were transcribed in Spanish, and analyzed using Framework Analysis [31, 32].

The codebook was reviewed by three researchers to ensure valid code development. Once the codebook was finalized by the research team, the transcripts were coded using *Dedoose* [33]. Following the steps of Framework Analysis, an analytic table was created to summarize each code across all transcripts. This analytic table allowed the team to systematically document the material and identify commonalities among the transcripts (refer to Additional file 1 for Codebook).

## Results

### Participant characteristics

A total of 10 promotores signed up for and participated in the eight-hour HBC training. Nine of the participants self-identified as female and one as male. All individuals were native Spanish speakers born in Latin America (Table 1). Most of the participants were not formally employed at the time of the training. Amongst the group, English proficiency ranged from limited knowledge to fluency. Around 40% of promotores previously attended trade school and 30% attended university. The average age of participants was 57, ranging from 32 to 69 years of age.

**Table 1:** The demographic data of the 10 promotores who attended the ‘Tu Historia Cuenta’ hereditary breast cancer training workshop

Characteristics	Mean (SD) or N (%)
Number of participants	10 (100)
Age, mean in years (SD)	51 (14)
Years in U.S, mean (SD)	23 (9)
English Language proficiency, N (%)	
Does not speak English	1 (10)
Knows enough basic words to get by	0 (0)
Can have a simple conversation	7 (70)
Completely fluent	2 (20)
Education level, N (%)	
Elementary	0 (0)
Middle School	1 (10)
High School	2 (20)
Trade School	4 (40)
University (4-year college)	3 (30)
Country of origin, N (%)	
Mexico	7 (70)
Peru	2 (20)
El Salvador	1 (10)
Employment status, N (%)	
Employed	4 (40)
Unemployed	6 (60)

### Pre- and post-workshop HBC training results

**Fig. 1:**
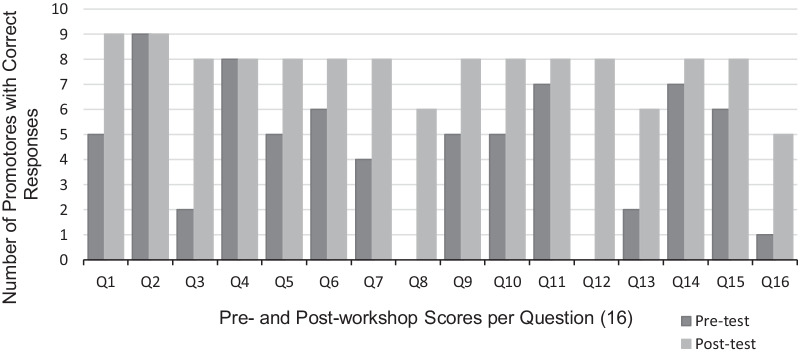
Number of correct answers in the pre- and post-hereditary breast cancer training workshop surveys by 9 participating promotores. Each survey included the same 16 questions (the questions are included in the Additional file 1)

One participant did not complete the pre- or post- survey and therefore, analyses included data from the nine promotores who completed both questionnaires. Results showed that individuals answered more questions correctly after the eight-hour workshop training compared to before the session (Fig. 1; refer to Additional file 1 for Survey Questions). The data showed an overall increase in correct responses after the training. Most progress was observed for questions regarding breast cancer characteristics (Q1 and Q3), genetic inheritance (Q7), genetic testing result interpretation (Q12), and BRCA mutations and prevention (Q8, Q13, and Q16).

### Qualitative findings

Our analysis of the pre- and post-session interviews revealed key facilitators in building promotores’ confidence and skills, as well as areas of ongoing challenge and recommendations.

## Facilitators

### Providing a supportive environment

The promotores highlighted their enjoyment working alongside the HBC investigators as it provided them with confidence to teach others about HBC. Additionally, individuals shared that working with the investigators made them feel valued, which encouraged high performance for every presentation. For instance, participant 1 recounted her experience:

“The first time [presenting], I felt very supported from you who are all very informed. That gave me a little bit of confidence. I did not feel… scared, nothing. I feel very satisfied.”

Participant 1 described how she felt prepared to respond to future questions her community may have due to the preparation, written materials, and guidance she had received. She felt confident in her role as a promotora to act as a liaison for the H/L population and assist them in navigating the health system.

### Practice presentations to prepare for community sessions

The promotores found the practice sessions helpful in improving and refining their presentations. Participant 2 who had previously served as a promotora, spoke positively of the practice sessions as it allowed her to incorporate the received feedback to her final presentation. She explained:

“[My presentation] got better because I realized what failed. It got better because I also saw my partner’s presentation and my own errors projected in her presentation. Also, because I am reviewing the material…. It is better because I realized what was not working for me and with her, and the form in which she was doing it. I said, ‘Oh there she is not doing well. That is not what they are expecting of us.’ Therefore, seeing my partner, provides me with feedback to see my own errors.”

Participant 2 continued to explain how the feedback provided her with a better understanding of the objectives of the community presentations and the team’s expectations. She also spoke of strategically focusing on challenging topics and incorporating the feedback to improve her presentations. Participant 1 had similar opinions regarding the practice sessions and described them as part of the preparation process.

### Promotores’ dedication and passion

The analysis revealed that a likely key factor in helping the promotores learn the HBC content was their passion for informing the H/L community. Participant 1 displayed her passion as she spoke of her past experiences as a promotora. She acknowledged the large-scale impact of her role as a promotora and elaborated on motivations from her community to perfect her presentation and ensure accurate transfer of knowledge. When asked how she felt leading her first practice session, she explained:

“I feel proud of myself. I believe that I can do it because I feel that I have the confidence to be in front of them [the community]. And over everything, I want to help them. I want them to understand that. I want that to be clear to them. I believe that we can save lives. And so that for me is something huge. That maybe a Latina, that did not know X information, did not know anything, but after they receive our information…That! That is what motivates me.”

Similar to participant 1, participant 2 has a longstanding passion for serving her community, which was the main driving factor for bettering her presentations. Participant 2 was clear about wanting the community to comprehend the valuable HBC information, and stressed the large responsibility and impact she can have on her community by stating:

“I am very happy with this project because it is a very big challenge that we promotores have to demonstrate to the researchers that we are capable…we are capable of transmitting it [the information] to the community. And if a Latina like them [the community] presents the program information in a more comprehensible way, there is more of an impact.”

Participant 2 expressed positive emotions in working with her community and the recognition of her work in the project. Her desire to improve her HBC knowledge and create a welcoming environment stems from her passion to serve as a role model for the H/L community.

## Recommendations for improvement

### Desire for flexibility when explaining HBC content

Both of the promotores expressed a desire for greater autonomy in delivering the academically rigorous topics using their own language and/or metaphors. Participant 1 communicated her reservations in informing the H/L community using scientific jargon. When asked if she had any modifications or advice to improve the program, she shared:

“My advice would be that they let us have influence because even though they do not believe us [referring to the supervisors of the program], we know where to stop [referring to their responsibility in only sharing HBC information they understand]. We know who we are with. And we know how to direct these people. So, yes, yes, we can. So that would be my advice, that they give us freedom…”

Participant 1 emphasized her determination to simplify the clinical language to ensure clear understanding. She stressed the importance of presenting the HBC content in a manner where an individual with limited educational background could comprehend the topics. Both individuals believed that with additional freedom and trust they could have a larger impact on the H/L population.

### Learning curve with the technological platform

The promotores found the management of the online platform to be the most challenging aspect of the program when conducting virtual practice education sessions in front of the research team. Individuals explained how their inexperience using Zoom created a challenge during practice sessions. Both promotores expressed greater comfort with face-to-face interaction and felt a lack of preparation to present with the virtual platform. Participant 1 shared her frustrations when asked if she felt that in-person presentations would be easier:

“Yes, of course. It is a more direct contact with the person. I feel that you pay more attention when the person is in front, training you. I see it that way. Besides, it is not easy to enter into all these thing (online platform). It is not easy. And much less giving a presentation when you have never had a computer in your life.”

She also feared that presentations held virtually would pose a barrier for many community members who do not know how to use online platforms. Both participants experienced similar challenges regarding the online presentations. In their opinion, the largest barrier for success was maneuvering the online system.

## Discussion

This qualitative study evaluated the promotores’ comfort and ability to lead HBC education sessions and modify the program for improvement. The increase in post-workshop HBC scores suggested that the HBC training enhanced the promotores’ knowledge concerning HBC and is consistent with our pilot training results [28]. The qualitative data found that through strong personal motivation and facilitator assistance (i.e., practice presentations, supportive environment), promotores can successfully discuss demanding topics like genetics. These findings support the use of promotores in future community-based interventions for complex subjects, as long as the program includes a strong training component to ensure accurate transfer of knowledge to the community.

The promotores expressed a positive experience with the program by highlighting the additional practice to review the HBC content, constructive feedback, and team support. These elements collectively led to a supportive program and should be utilized as a reference when developing future projects. Promotores’ limited background knowledge of HBC did not prevent them from presenting the materials clearly and confidently after appropriate training. Working alongside the researchers provided them with the courage and confidence to continue perfecting their presentations and understanding of HBC. These findings are supported by the robust literature suggesting the efficacy of community-based programs and how successful interventions are attributed to a supportive team [34–36] This data adds to the already existing large body of literature demonstrating promotores as effective health educators [21, 24, 37]. Findings support scaling up promotor programs to disseminate health knowledge to vulnerable populations.

Promotores’ motivation to serve the H/L community and desire to disseminate correct HBC knowledge influenced their success in the program. Although learning the HBC content was found difficult at first, the promotores remained dedicated. Aligned with existing literature, motivation played a key factor in the promotores' success and retention in the HBC program [36]. Current literature indicates that retention is a challenge in promotor-based programs [38–40]. Recommendations include screening promotores for strong leadership qualities, strong ties to the community, and providing payment for their time [38]. To ensure high retention rates among promotores in the HBC program, we should continue prioritizing individuals who are highly motivated and eager to positively impact the H/L community.

The unexpected transformation of the program into an online-platform provided an opportunity to learn about the challenges associated with implementing virtual learning options for the underserved Spanish-speaking H/L community. Both promotores believed that unfamiliarity with the technological aspect of the virtual sessions posed a great challenge to sharing the HBC information.

This key finding provided insight into modification to improve the program, such as additional training with the online applications. Given that COVID-19 has transformed many research interventions into virtual formats, it is essential to consider and address potential barriers. For instance, financial barriers may prevent target populations from accessing high speed internet or a smartphone. When developing novel interventions or adapting pre-existing ones to an online format, additional thought should be put into removing obstacles faced in virtual education.

The continuous support from the research team and community organization beyond training and during implementation would be important for promotores as they confront potential logistical barriers or are phased with complex questions that may arise from the community. A plan for ongoing support will ensure the promotores have the resources to share information successfully and facilitate transfer of knowledge to the community.

## Conclusion

Overall, findings suggest that the interplay between the practice sessions, supportive environment, and motivation to serve the H/L community were key factors in the participant’s confidence and ability to explain HBC content. The project aimed to discover what modifications were needed to improve the fidelity and confidence with which promotores informed the community about HBC. Observing the participants’ progress over an eight-week period allowed researchers to gain insight on the elements of a successful program and areas for improvement. Conducting multiple interviews with promotores was a strength of the study as it provided in-depth data regarding promotores’ perspectives and experiences. Although the sample size was limited, the data can help inform future interventions as well as improve the HBC community-based education program as it continues to expand across California.

## Supplementary Information

**Additional file 1.** Supplementary materials including (S1) pre and post promotores workshop survey, (S2) pre and post promotores virtual training session interview guides, (S3) promotores interviews qualitative analysis code book.

## Data Availability

All de-identified data generated during this study (e.g., interview transcripts, observation notes, survey responses) are available from the authors upon request.

## Abbreviations

HBC: Hereditary Breast Cancer
H/L: Hispanic/Latinx
U.S.: United States
TLCI: Latino Cancer Institute
UCSF: University of California, San Francisco
