# Supplementary material for: Training promotores to lead virtual hereditary breast cancer education sessions for Spanish-speaking individuals of Latin American heritage in California
Source: BMC Womens Health. 2022 Aug 8;22:336. doi: 10.1186/s12905-022-01902-y (PMC9358079; doi:10.1186/s12905-022-01902-y)
Supplement: Supplementary file 1 — Additional file 1. Supplementary materials including (S1) pre and post promotores workshop survey, (S2) pre and post promotores virtual training session interview guides, (S3) promotores interviews qualitative analysis code book. [file 12905_2022_1902_MOESM1_ESM.docx]

**Supplementary Materials**

Pre and Post- Workshop Survey

**1. Cancer is characterized as**
❏ Uncontrolled cell division

❏ controlled cell division

**2. Tumors are always malignant.**

❏ True

❏ False

**3. Stage describes how advanced the cancer is**

❏ Yes

❏ No

**4. The earlier you identify breast cancer, the greater the chances of treatment and survival**

❏ True

❏ False

**5. A gene is**
❏ It is like a page of instructions that tell the body how to work

❏ It is very small and therefore not important in cancer risk

**6.A mutation is a change in the genetic material.**

❏ True

❏ False

**7. Babies inherit half of their genetic material from their mother and the other half from their father.**

❏ True

❏ False

**8. Which statement is correct about the BRCA 1 and BRCA 2 genes?**

❏ All humans have BRCA 1 and 2 genes. Mutations in these genes can increase your risk of breast cancer.

❏ BRCA 1 and 2 genes cause breast cancer

❏ Only Latina women have BRCA 1 and 2 genes

**9. It is important to do a genetic test when you have a history of breast cancer in your family because the test tells us about the probability of developing breast cancer and also tells us if our children or grandchildren have a higher probability.**

❏ True

❏ False

**10. Who should talk with a genetic counselor?**

❏ People with a family history of breast cancer

❏ People who are older

❏ People who have unhealthy lifestyles

**11. If my brother’s wife and my aunt’s husband have cancer, does that mean that my risk of developing breast cancer is higher than any other woman?**

❏ Yes

❏ No

**12.A_____ result for genetic testing of BRCA 1 and 2 indicates that the person has inherited a harmful mutation.**

❏ Positive

❏ Negative

**13. What are some ways to reduce your risk of breast cancer?**

❏ Breastfeed for long periods
❏ Regular physical activity
❏ Eat healthy
❏ All of the above help reduce the risk of breast cancer

**14. Any women can get breast cancer, even if no one in their family has it.**

❏ True

❏ False

**15.A genetic test is done with a blood or saliva sample.**

❏ Yes
❏ No

**16. How can someone with a harmful BRCA 1 and 2 mutation control their cancer risk?**

❏ Intensified screening

❏ Preventative surgery (to reduce risk)

❏ Chemoprophylaxis

❏ All of the options mentioned help control the risk of breast cancer if you have a harmful BRCA 1 and 2 mutation.

**Pre-session Interview Guide**

1. Can you tell me a little bit about your background and why you became a promotor/a?

What is it about being a promotor/a do you like?

2. Did you enjoy the hereditary breast cancer program?

What parts were more excited and you enjoyed the most

Where there any parts that you did not enjoy/ found boring?

3. Were there any topics you felt easy to comprehend?

What topics did you felt were more challenging?

4. What about those topics (specific) made learning them challenging?

5. What topics do you think you understood the best and why?

6. After taking the workshop, do you feel prepared to teach community members about hereditary breast cancer and answer questions community members may have?

7. What concepts that you learned during the hereditary breast cancer education program are you most confident in teaching?

8. What concepts of the hereditary breast cancer education program are you the most nervous to teach?

9. Do you think that the hereditary breast cancer education program prepared you well enough to begin workshops within the community?

10. Would you change any part of the hereditary breast cancer education program?

If yes, what would you add or change?

**Post-session Interview Guide**

1. How are you doing since the last time we talked?

2. How did the hereditary breast cancer education workshop go?

Did you feel comfortable?

3. Were there any topics in the hereditary breast cancer program you found difficult while teaching?

4. I saw you did really well in describing (topic they did well in). How did you feel about it?

5. When describing (topic they struggled with) how did you feel about it?

6. Did you feel confident with you overall performance?

7. Were there any specific parts of the workshop you felt very confident teaching?

8. Did you find that you had enough information to answer the community members questions?

9. Has participating in the hereditary breast cancer education program as a promotora changes your feelings about yourself?

10. For the next workshop are you planning to do anything differently?

**Code Book**

| Codes | Descriptions |
| --- | --- |
| Motivations/inspirations | Personal motivations for wanting to join the HBC program, personal values and goals that lead them to help others, what inspires them to serve as promotores |
| Barriers to presenting | This includes barriers such as limited internet connection, unfamiliar with online systems such as ZOOM, unfamiliar with computers, anything not related to learning the topics, it may also include barriers the participants could face when accessing the presentation |
| Barriers to learning | Information regarding why the promotores had a hard time learning some of the HBC topics, barriers that they hard to overcome when learning |
| Topics comfortable/confident presenting | These are topics the promotores fell comfortable discussing/ presenting which include genetics and non-genetics topics |
| Topics nervous/difficult presenting | These are topics the promotores felt nervous to present or topics they felt they struggled to present, this includes genetic and non-genetics topics |
| Previous/outside work experience | This involves any work experience the promotores were involved in before the HBC project, it also involves any additional work they are doing alongside the HBC program |
| Preparation | This includes any descriptions about how they are preparing for their practice presentations such as note taking, practicing with virtual platform, rehearsal, watching their partner present the information, outside knowledge that has helped improve their presentation skills |
| Advice/ modifications | This includes any information the promotores provided on how to improve the program, practice presentations, mode of knowledge transfer |
| Personal information | Any personal information not relevant to the HBC program, could be personal information about their family |
| Quotes quotable | Quotes important for analysis |
| Feelings toward the project/presentation | Describes the promotores feelings about their practice presentations, do they feel like they improved, how prepared they feel about future presentations, goals they set for future presentations  This also includes the promotores feelings toward the HBC program as a whole, how it is run, how they felt the practice presentations helped their presentations, the amount of support they were given |
| Topics important for the community | Topics the promtores found relevant/ interested in learning/topics they enjoyed learning  These are topics that the promotores feel are important to relay to the community, topics they would like to see the community understand, and topics that they believe the community would benefit from the most |
| Promotores role | The promotores understanding the role they play within the community/HBC program. Promotores self-perceptions: meaning they felt more important to society, captures changes in self-image over the course of the practice presentations, why they believe promotores are important, and their impact with within the community. |
